# MetabR: an R script for linear model analysis of quantitative metabolomic data

**DOI:** 10.1186/1756-0500-5-596

**Published:** 2012-10-30

**Authors:** Ben Ernest, Jessica R Gooding, Shawn R Campagna, Arnold M Saxton, Brynn H Voy

**Affiliations:** 1 Graduate School of Genome Science and Technology, University of Tennessee, Knoxville, TN 37996, USA; 2 Department of Animal Science, University of Tennessee, Knoxville, TN 37996, USA; 3 Department of Chemistry, University of Tennessee, Knoxville, TN, 37996, USA

**Keywords:** R script, User-friendly, Linear mixed model, Statistics, Normalization, Mass spectrometry-based metabolomics

## Abstract

**Background:**

Metabolomics is an emerging high-throughput approach to systems biology, but data analysis tools are lacking compared to other systems level disciplines such as transcriptomics and proteomics. Metabolomic data analysis requires a normalization step to remove systematic effects of confounding variables on metabolite measurements. Current tools may not correctly normalize every metabolite when the relationships between each metabolite quantity and fixed-effect confounding variables are different, or for the effects of random-effect confounding variables. Linear mixed models, an established methodology in the microarray literature, offer a standardized and flexible approach for removing the effects of fixed- and random-effect confounding variables from metabolomic data.

**Findings:**

Here we present a simple menu-driven program, “MetabR”, designed to aid researchers with no programming background in statistical analysis of metabolomic data. Written in the open-source statistical programming language R, MetabR implements linear mixed models to normalize metabolomic data and analysis of variance (ANOVA) to test treatment differences. MetabR exports normalized data, checks statistical model assumptions, identifies differentially abundant metabolites, and produces output files to help with data interpretation. Example data are provided to illustrate normalization for common confounding variables and to demonstrate the utility of the MetabR program.

**Conclusions:**

We developed MetabR as a simple and user-friendly tool for implementing linear mixed model-based normalization and statistical analysis of targeted metabolomic data, which helps to fill a lack of available data analysis tools in this field. The program, user guide, example data, and any future news or updates related to the program may be found at http://metabr.r-forge.r-project.org/.

## Findings

### Background

Quantitative metabolomics is a high-throughput approach to systems biology in which many small molecules (metabolites) from a biological sample are simultaneously measured, commonly using nuclear magnetic resonance spectroscopy (NMR), gas chromatography—mass spectrometry (GC-MS), or liquid chromatography—mass spectrometry (LC-MS). While transcriptomics and proteomics are established approaches for characterizing the effects of experimental conditions on metabolism, gene and protein expression changes merely indicate the potential for changes in metabolic endpoints. Metabolic changes are “real-world” endpoints, so metabolomics can connect these functional genomics platforms with actual physiology [1].

LC-MS metabolomic approaches fall into two categories: those that attempt to measure every metabolite in the sample (untargeted approaches) and those that attempt to measure only a subset of the metabolites (targeted approaches) [2]. A key benefit of targeted approaches is that the detected metabolites can also be readily quantified. Like other approaches to systems biology that rely on the analysis of multiple samples to generate large datasets, two important issues hold true in targeted metabolomics. First, experiments frequently are carried out in multiple “blocks”. For example, targeted LC-MS metabolomic platforms involve lengthy instrumental runs and may rely on multiple runs to enhance metabolite coverage [3,4], often necessitating multiple run days to analyze all samples. Each run day represents a different block, which introduces technical variability in metabolite detection signals from day-to-day variances in factors related to the instrument’s operation, such as injection volume and ionization efficiency. Second, sampling and measurement variables introduce technical variability in metabolite detection signals, including tissue mass (for multicellular organisms), cell number and size (for microorganisms), sample matrix effects, and mass spectrometer variability (measured by the signal from an internal standard present in the metabolite extraction solvent in our experiments). Clearly, the metabolite signal variability due to block and sampling/measurement variables needs to be distinguished from variability due to experimental treatment factors, which calls for a normalization step to remove the effects of such confounding variables.

Conventional LC-MS metabolomic data normalization is carried out by expressing each metabolite signal relative to values of sampling/measurement variables [3,4]. Statistical tests for mean differences between treatment groups are performed on normalized metabolite values, with metabolite means averaged across the levels of any block factors (i.e., run day).

There are limitations to this conventional normalization approach, however. First, often many metabolites are normalized to one internal standard (i.e., one for all positive ions and one for all negative ions). This would introduce additional bias if there were low or negative correlation between the internal standard signal and a metabolite signal (i.e., for metabolites with different chemical properties from the internal standard), or if the internal standard signal differed significantly between treatment groups. Second, while ignoring block factors (i.e., comparing metabolite means averaged across samples analyzed on different days) increases sample size, significant block effects on metabolite signals may widen confidence intervals, which may preclude identification of “significant” metabolites and conceal statistical outliers. Block effects may dramatically bias the data, especially if they are not balanced across treatment groups.

Currently available software packages provide powerful tools for pre-processing (i.e., peak selection and integration and retention time alignment), visualization (i.e., biochemical pathway mapping), and/or interpretation of targeted and untargeted metabolomic data [5-10]. However, these packages have limitations because they either 1) do not provide normalization tools for removing confounding effects of experimental variables [7-9]; 2) use the conventional normalization approach [6]; or 3) require the researcher to manually determine a normalization factor for each experimental sample [5].

A flexible and standardized normalization approach that improves on current limitations would improve metabolomic analyses. An efficient and intuitive approach to control for confounding variables is to estimate their effects on metabolite signals using linear models. Rather than assuming similar relationships between each metabolite signal and confounding variables, a linear model fit for each metabolite can be used to estimate and partition the effects of each experimental variable, including treatment factor, on each metabolite signal. Further, experimental variables can be modeled as having either a fixed or random effect on metabolite signals, with important implications. Fixed-effect variables are assumed to have a constant effect on metabolite signals, influence metabolite signals in an anticipated direction, and have a similar influence in replicate experiments. Common fixed-effect variables are number of cells, tissue mass, and ionization efficiency. By comparison, the effects of random-effect variables cannot be anticipated *a priori*, and they create variation, but overall do not influence metabolite signals. Typical examples are specimen gender, species or line, experiment day, instrument, and technician [11], although some of these could be treated as hypothesis-driven experimental factors in some experiments.

Mixed models can be used to estimate the effects of fixed- and random-effect variables on a response variable [11] and are an established approach for normalizing microarray data [12-21]. For two primary reasons, however, currently available microarray data normalization tools are not suitable for metabolomic data. First, microarray normalization tools adjust data for systematic effects specific to microarray technology, such as “dye bias” of different fluors, spatial position effects on the microarray chip, background signals, and biases due to probe binding strengths [22]. Second, microarray normalization tools are often platform specific, designed to carry out pre-processing and quality control only for Illumina BeadArray or for Affymetrix GeneChip platforms, for example [23].

Given the limitations of current metabolomic data normalization approaches, we developed MetabR, a simple, user-friendly, and stand-alone tool that researchers with no programming background can use to implement linear model-based normalization and statistical analysis of targeted metabolomic data downstream of pre-processing. While MetabR is stand-alone, software with pre-processing tools [5,6,8] can be used to generate the input data for MetabR. Further, MetabR generates output files that may be used in subsequent analysis, including normalized data, a heat map and dendrogram, and a comma-separated values (CSV) file formatted for direct upload into Pathway Projector [9], a web-based biochemical pathway visualization tool.

### Methods

#### Implementation of MetabR

**Figure 1:**
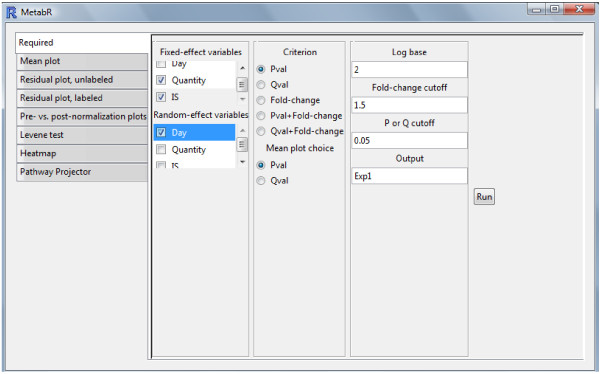
Screenshot of the MetabR GUI.

A graphical user interface (GUI)-based program, MetabR (Additional file 1), was written in the R open-source language (version 2.15). A screenshot of the GUI is shown in Figure 1. The GUI was built using the “gWidgets” package [24]. As described in the user guide (Additional file 2), the GUI is used to select which variables to define in a normalization model as fixed- and random-effect variables and to tailor statistical analysis to the researcher’s needs. As a threshold to screen for metabolites that differ significantly in abundance between treatment groups, the researcher may choose p-value, q-value, mean fold-change, or a combination of p-value or q-value and mean fold-change, as well as the specific values of these thresholds.

In this program, either a fixed linear model (function “lm” in the “stats” package) or a linear mixed model (function “lmer” in the “lme4” package) [25] is constructed that includes the normalization variables selected by the user, and one Group treatment factor, for example

Metabolite=μ+Group+β∗Quantity+β*IS+Day+e,

where μ = group mean,

Group = treatment factor,

Quantity = a measured, continuous value of the amount of tissue used to produce each sample,

IS = a measured, continuous value of the detection signal from an internal standard present in the metabolite extraction solvent,

Day = a normalization factor accounting for the effects of different run days on metabolite signals,

and e = residual error.

The residuals and treatment group means from the fitted model are added together to yield normalized data, which adjusts for effects of sample quantity, ionization efficiency, and run day, as appropriate for the experimental design of the study.

To check normality and equal variance assumptions made by linear models, R functions “shapiro.test” in the “stats” package (“stats” and any other packages not referenced are part of R [26]) and “levene.test” in the “lawstat” package [27] are used, respectively. In addition, residual error plots are produced, and normalized data are exported for possible secondary use by the researcher. Tukey’s Honest Significant Difference (HSD; function “TukeyHSD” in the “stats” package) method is used to test for treatment group mean differences in the normalized data based on the Studentized range statistic. Q-values [28] are calculated from the list of Tukey HSD p-values for each treatment group comparison using the “qvalue” function in the “qvalue” package [28]. If any treatment group mean is significantly different from any other, a group mean plot with confidence intervals is constructed for the metabolite. Differences among treatment group means are represented by letter groupings generated by code adapted from a SAS macro [29], with means that share any letter being statistically equal. Further, statistical results from “significant” metabolites are exported into a spreadsheet that can be directly uploaded to Pathway Projector [9], which uses the information to map the metabolites, colored dots representing the direction and size of mean fold-changes, and either p- or q-values, to biochemical pathways. The program generates a series of files listed in Table 1 and described in the user guide (Additional file 2).

**Table 1:** Output files produced by the MetabR program

**Output**	**File type**
Normalized data	CSV
Normalized data with technical replicates averaged	CSV
A plot of the model residuals for each metabolite vs. each metabolite’s overall mean signal	PDF
A plot of the model residuals for each metabolite vs. each metabolite’s overall mean signal, expanded to accommodate metabolite labels	PDF
Mean plots for all significant metabolites	CSV
Tukey HSD p-values for all treatment group comparisons for every metabolite	CSV
q-values for all treatment group comparisons for every metabolite	CSV
Mean fold-changes between all treatment group comparisons for every metabolite	CSV
Plots of all confounding variables vs. all metabolite measurements, pre- and post-normalization	PDF
Heat map and dendrogram of the normalized data	PDF
Spreadsheet for direct upload to Pathway Projector	CSV

#### Experimental data collection

Two experimental datasets were generated in our lab to illustrate the utility of MetabR. In both experiments, adipose tissue samples were flash frozen in liquid nitrogen and powdered with a mortar and pestle before metabolite extraction, which followed a previously described procedure [30]. The extracted metabolomes were then analyzed by liquid chromatography—tandem mass spectrometry (LC-MS/MS) via a slightly modified version of the methods of Rabinowitz and co-workers [30-32] that scans for approximately 350 total metabolites in positive and negative ionization modes. The Quan Browser function in the Xcalibur software package (Thermo Scientific, Waltham, MA) was used to assess the presence of each metabolite based on standard detection parameters, such as retention time, signal-to-noise ratio, and peak shape. Signal intensity in ion counts was then determined using Xcalibur to manually integrate each peak, and these data were exported into a Microsoft Excel spreadsheet for statistical analysis.

The first experiment was designed to examine the effects of dietary restriction and insulin immunoneutralization on adipose tissue metabolism in chickens. A total of 127 metabolites were detected in abdominal adipose tissue from 16- or 17-day-old male broiler chicks that were fed *ad libitum* (“Control”), fasted for 5 hours (“Fast”), or immunoneutralized against the effects of endogenous insulin (“InsNeut”), as we previously described [33,34]. This study included two factors, Treatment and Day (day 1, day 2, or day 3). Fourteen metabolite measurements from this experiment are provided in Additional files 3 (“Chicken example data 1”) and 4 (“Chicken example data 2”), corresponding to metabolites detected in positive and negative ionization modes, respectively.

The second experiment was designed to examine the effects of Bisphenol A (BPA) on adipose tissue metabolism in mice. A total of 93 metabolites were detected in abdominal adipose tissue from 32 16-week-old inbred male mice which, from weaning, were fed *ad libitum* and given drinking water spiked with 0, 0.05, 0.5, or 5 μM BPA. Sixteen mice from each of the inbred strains C57BL/6J and DBA/2J were used in this study. A few missing values arose when a metabolite was not detected in a subset of the samples. Using a zero value for these measurements would bias the results, so they were set to missing (“NA”) which excludes that measurement from analysis. This study included three factors, Treatment, Strain (C57BL/6J or DBA/2J), and Day (day 1, day 2, day 3, or day 4). Twelve metabolite measurements from this experiment are provided in Additional files 5 (“Mouse example data 1”) and 6 (“Mouse example data 2”), corresponding to metabolites detected in positive and negative ionization modes, respectively.

#### Modeling confounding variables as fixed- vs. random-effect

In our chicken example, Group, Quantity, and IS were modeled as fixed-effect variables, while Day was modeled as a random-effect variable. To illustrate the difference, if Day is defined as a fixed-effect variable, the estimated treatment group mean includes the average Day effects, and the variance and corresponding confidence intervals are based only on residual error and sample size. Inferences about treatment effects refer only to the days used in the experiment. If Day is defined as a random-effect variable, the estimated mean no longer includes Day. Instead, the Day effect becomes a source of random variation that is added to the variance of the estimated mean. The variance and confidence intervals are larger than those when Day is treated as a fixed-effect variable, but experimental results can now be correctly extrapolated to all possible days [11].

### Results

#### Chicken experimental results

For the chicken data, Quantity (tissue mass) and IS (internal standard measurement, Tris in positive ionization mode and Benzoic Acid in negative ionization mode) were selected as fixed-effect regression variables, and Day (run day) as a random-effect factor.

Summary information printed in the R console (not shown) includes 1) results from the Shapiro-Wilk test of normality; 2) results from Levene’s test of equality of variance; 3) pairwise mean fold-changes between all treatment groups for significant metabolites (also exported into a spreadsheet; see Table 2 for the data); and 4) pairwise Tukey HSD p-values or q-values between all treatment groups for significant metabolites (also exported into a spreadsheet; see Table 2 for the data). This printout showed that Shapiro-Wilk p-values for all metabolites were greater than 0.05, indicating no violation of the assumption of normality. Levene’s test p-values for Citraconate and Inosine were less than 0.05, indicating a possible violation of the linear model assumption of equality of variance. By using the diagnostic results from Shapiro-Wilk and Levene’s tests, researchers can identify when data are unacceptable for use with the linear model approach. Ion counts are sometimes modeled as Poisson distributed, so if normality concerns are still an issue after opting for a log transformation, the researcher may wish to pursue an alternative statistical approach.

**Table 2:** Chicken experiment fold-changes

	**Treatment comparison**					
	**Fast-control**		**InsNeut-control**		**InsNeut-fast**	
**Metabolite**	**Fold-change**	**P-value**	**Fold-change**	**P-value**	**Fold-change**	**P-value**
ATP	1.273	0.384	1.059	0.932	0.832	0.588
Citraconate	0.969	0.694	0.982	0.915	1.014	0.907
Citrate	1.251	**0.047**	1.054	0.720	0.842	0.196
Dihexose	0.082	**<0.001**	0.590	0.928	7.217	**0.001**
Inosine	0.736	0.328	0.910	0.580	1.236	0.890
Lactate	0.873	0.137	0.991	0.974	1.135	0.198
Pyruvate	1.100	0.353	1.065	0.640	0.969	0.870
2-Oxoglutarate	0.929	0.754	1.511	**0.001**	1.627	**<0.001**
1-Methyladenosine	0.934	0.878	0.923	0.865	0.989	1.000
Glutamine	0.676	**0.026**	2.512	**<0.001**	3.715	**<0.001**
Guanosine	0.762	0.215	0.833	0.257	1.094	0.993
O-Acetyl-L-serine	0.614	0.337	2.276	0.085	3.707	**0.004**
Glucosamine	1.014	0.959	2.073	**<0.001**	2.044	**<0.001**
Thiamine	0.486	0.059	0.781	0.860	1.607	0.156

Mean fold-changes among the three treatment groups for the chicken example data (14 metabolites across positive and negative ionization modes), and associated Tukey HSD p-values for mean differences (bold values are p < 0.05).

**Figure 2:**
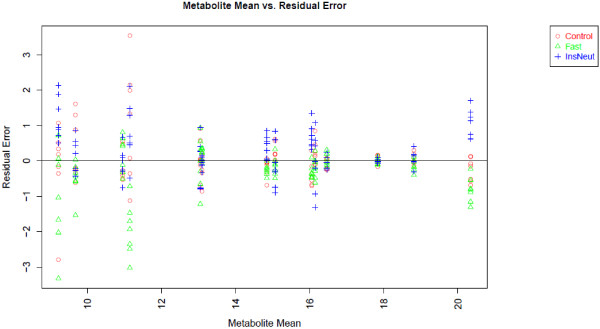
**Residual error plot for the chicken experiment.** Legend - Linear model residuals are plotted in relation to overall mean metabolite level.

Figure 2 contains the plot of residual error for each metabolite after data transformation and normalization in relation to the overall mean abundance for each metabolite across all samples (we used log base 2 transformation). This plot can be used to determine whether data transformation and normalization corrected for the typical relationship of increasing variance with increasing mean. In this example, variance is visually relatively consistent across groups, and somewhat greater for low-abundance metabolites.

**Figure 3:**
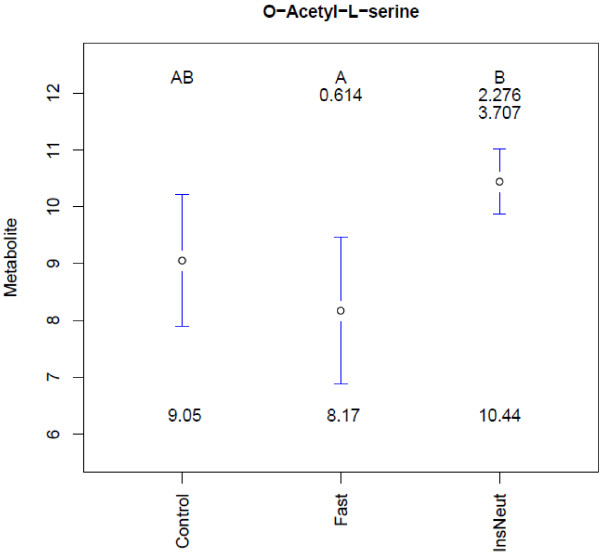
**Group mean plots for *O *-Acetyl-l-serine in the chicken experiment.** Legend - Treatment group metabolite means, 95% confidence intervals, mean fold-changes, and significant difference letters are combined to summarize results for each significant metabolite.

Figure 3 illustrates an example of the mean plots and 95% confidence interval bars created for metabolites with a statistically significant effect of treatment. *O*-Acetyl-l-serine levels were significantly lower in Fast samples compared to InsNeut. Mean separation letters indicate that Fast and InsNeut groups differed significantly from each other (p < 0.05 threshold chosen), but neither differed from Control. Fold-changes between treatment group means (not log transformed) are displayed below the letters. Fold-changes in the *n*th row correspond to comparisons with the group in the *n*th column, (i.e., the mean of *O*-Acetyl-l-serine was 3.707-fold higher in InsNeut compared to Fast).

**Figure 4:**
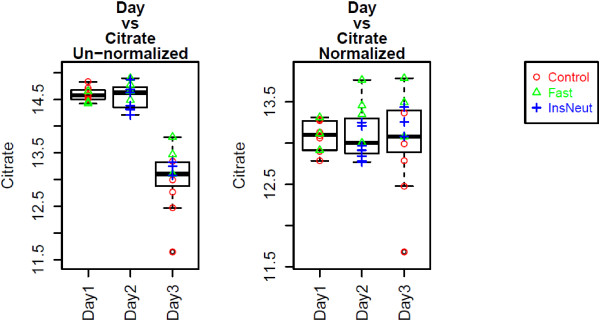
**Pre- and post-normalization plots: metabolite vs. Day.** Legend - Citrate is plotted before and after normalization, showing the effectiveness of the normalization model for removing confounding variation in the chicken experiment. Normalization removed the effect of different run days on the Citrate detection signal.

**Figure 5:**
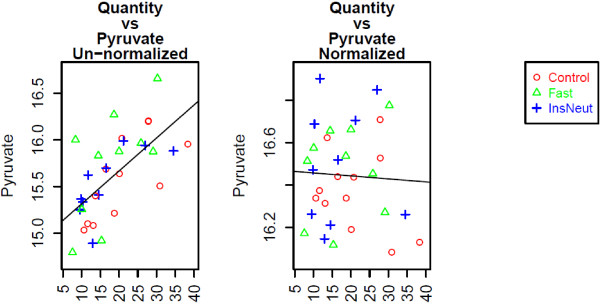
**Pre- and post-normalization plots: metabolite vs. tissue quantity.** Legend - Normalization removed the correlation between the quantity of tissue analyzed and the Pyruvate detection signal in the chicken experiment.

Figure 4 shows a box-and-whisker plot of Citrate vs. run day, an ANOVA confounding variable, before and after data normalization with MetabR. Figure 5 shows a scatter plot of Pyruvate vs. Quantity, a regression confounding variable, before and after data normalization with MetabR. These plots are produced automatically by MetabR for all metabolites and all confounding variables included in the input data, and they give visual verification that the effects of confounding variables on metabolite measurements were removed by normalization using the linear model approach.

**Figure 6:**
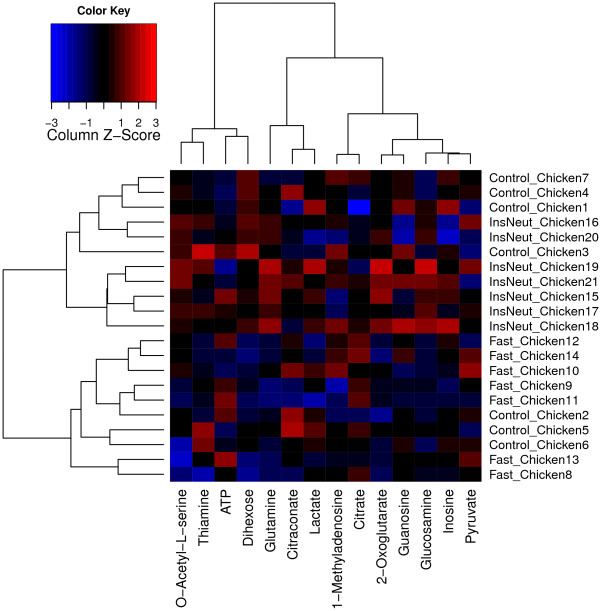
**Heat map and dendrogram.** Legend - The heat map was produced by the MetabR program using the chicken example data included in Additional files 3 and 4. The metabolites are in the columns and the chicken adipose samples are in the rows. Columns are mean-centered, with relative abundance represented by color (blue, lower abundance; red, higher abundance), as indicated in the legend. InsNeut chickens cluster in the upper half of the dendrogram, completely separate from Fast chickens, suggesting that these two treatment groups have distinct metabolic signatures, while the metabolic signature of the Control chickens appears less distinct. Note: the LC-MS/MS instrument method is unable to differentiate between the several isomeric dihexoses, and therefore they are measured as a group.

Figure 6 shows a heat map and dendrogram of the normalized data, produced automatically by MetabR via the “heatmap.2” function from the “gplots” package [35]. A heat map is useful for visualizing overall differences in metabolic signatures, and the dendrogram gives visual evidence of whether the experimental conditions significantly influenced metabolic signatures. Each metabolite plotted is mean-centered, helping to call attention to metabolites differing in abundance among samples. The chickens appear to cluster non-randomly based on their overall metabolic signatures (Figure 6). InsNeut chickens cluster in the upper half of the dendrogram, completely separate from Fast chickens, suggesting that these two treatment groups have distinct metabolic signatures, while the metabolic signature of the Control chickens appears less distinct.

Table 2 contains all between-group mean fold-changes for the metabolites, with differences tested by Tukey’s HSD at the 5% significance level. We produced this table by combining the mean fold-changes and p-values exported automatically by MetabR. As shown, the experiment had sufficient power to detect a fold-change as low as 1.25 for Citrate between Fast and Control groups. In general, the differences between the Control and InsNeut groups were smaller than other treatment group comparisons. The program exports q-values automatically, and the researcher may select p-value, q-value, mean fold-change, or a combination of either p-value or q-value and mean fold-change as a significance threshold. As technological improvements continue to allow more metabolites to be detected, the chance of false discoveries will increase, making false discovery corrections (q-value) increasingly necessary.

#### Mouse experimental results

MetabR was run on the mouse example data in Additional files 5 and 6, selecting the same parameters as the chicken experiment, except that “Strain” (C57BL/6J or DBA/2J) was additionally selected as a random-effect variable in order to remove the effects of different mouse strains on metabolite measurements. Two metabolites had Shapiro-Wilk p-values less than 0.05 and W statistics less than 0.90, indicating possible violations of normality. No metabolites were identified as having unequal variance among treatment groups. The residual plot (not shown) also showed no evidence of unequal variance, and it was visually apparent that variance was equal across all measurement levels, and thus the log base 2 transformation chosen for the analysis was effective. Fold-change results are given in Table 3.

**Table 3:** Mouse experiment fold-changes

	**Treatment comparison**											
	**BPA500-BPA50**		**BPA5000-BPA50**		**Control-BPA50**		**BPA5000-BPA500**		**Control-BPA500**		**Control-BPA5000**	
**Metabolite**	**Fold-change**	**P-value**	**Fold-change**	**P-value**	**Fold-change**	**P-value**	**Fold-change**	**P-value**	**Fold-change**	**P-value**	**Fold-change**	**P-value**
Bisphenol A	0.817	0.998	0.455	0.423	1.420	0.984	0.558	0.490	1.738	0.946	3.117	0.261
Glucose-6-phosphate	1.042	0.081	0.987	0.859	1.023	0.545	0.947	**0.013**	0.981	0.654	1.036	0.168
Lactate	1.663	0.298	1.177	0.923	1.353	0.401	0.708	0.652	0.814	0.997	1.149	0.771
Citrate	1.064	1.000	3.265	0.120	2.273	0.219	3.070	0.141	2.137	0.252	0.696	0.988
Isocitrate	0.809	0.219	1.134	0.644	1.117	0.731	1.401	**0.019**	1.380	**0.026**	0.985	0.999
Phosphoenolpyruvate	1.218	0.551	1.476	0.167	0.793	0.962	1.212	0.852	0.651	0.287	0.537	0.064
Thymine	0.868	0.919	0.552	**0.025**	1.118	0.972	0.636	0.100	1.288	0.710	2.026	**0.009**
Urea	1.325	0.971	0.960	0.993	1.084	0.947	0.725	0.894	0.818	1.000	1.129	0.849
N-Acetyl-L-glutamate	0.449	**0.001**	0.518	**0.007**	0.548	**0.014**	1.152	0.789	1.220	0.638	1.059	0.994
ADP	1.264	0.907	7.812	0.092	11.948	0.035	6.180	0.280	9.452	0.124	1.530	0.957
Tryptophan	1.086	0.998	0.757	0.461	0.870	0.912	0.697	0.367	0.801	0.841	1.150	0.843
Ornithine	1.813	**0.008**	1.563	0.071	1.231	0.476	0.862	0.776	0.679	0.189	0.788	0.686

Mean fold-changes among the four treatment groups for the mouse example data (12 metabolites across positive and negative ionization modes), and associated Tukey HSD p-values for mean differences (bold values are p < 0.05).

### Conclusions

The open-source statistical computing software R [26] provides a convenient environment for statistical analysis of metabolomic and other -omic data. We developed a user-friendly R program that normalizes metabolomic data using linear mixed-effect modeling (with regression and ANOVA terms), statistically compares treatments, and exports results files to aid data interpretation, filling an important lack in statistical analysis tools available to the metabolomics community. The MetabR program file, example data, and user guide are available as an R-Forge project at http://metabr.r-forge.r-project.org/. This website will also contain future news or updates related to MetabR, including availability through the Comprehensive R Archive Network (CRAN) or Bioconductor.

## Availability and requirements

**Project name:** MetabR

**Project home page:**http://metabr.r-forge.r-project.org/

**Operating system(s):** Windows, Mac, Linux, any system that runs R

**Programming language:** R

**Other requirements:** Required R packages are installed automatically. The program was written and tested using R version 2.15 for Windows.

**License:** GNU General Public License (GPL)

**Any restrictions to use by non-academics:** No restrictions

## Availability of supporting data

The datasets supporting the results of this article are included within the article (and its additional files).

## Abbreviations

ANOVA: Analysis of variance; BPA: Bisphenol A; CSV: Comma-separated values; GUI: Graphical user interface; HSD: Honest Significant Difference; IS: Internal standard; LC-MS: liquid chromatography—mass spectrometry; LC-MS/MS: Liquid chromatography—tandem mass spectrometry.

## Competing interests

The authors declare that they have no competing interests.

## Authors’ contributions

BE wrote the program. BE, SRC, BHV, and JRG collaborated to outline the issues in data analysis and process all biological data. AMS guided implementation of the statistical analysis components of the program. JRG and BE tested the implementation of the program, and all authors contributed to writing the final manuscript draft. All authors read and approved the final manuscript.

## Authors’ information

J Gooding’s current address: Sarah W. Stedman Nutrition & Metabolism Center, Duke University School of Medicine, 4321 Medical Park Drive, Suite 200, Durham, NC 27704

## Supplementary Material

Additional file 1 **MetabR.** MetabR program file. Click here for file

Additional file 2 **User Guide.** MetabR user guide. Click here for file

Additional file 3 **Chicken_pos.** Chicken example data 1 from positive ionization mode. Click here for file

Additional file 4 **Chicken_neg.** Chicken example data 2 from negative ionization mode. Click here for file

Additional file 5 **Mouse_pos.** Mouse example data 1 from positive ionization mode. Click here for file

Additional file 6 **Mouse_neg.** Mouse example data 2 from negative ionization mode. Click here for file
