# Supplementary material for: MetabR: an R script for linear model analysis of quantitative metabolomic data
Source: BMC Res Notes. 2012 Oct 30;5:596. doi: 10.1186/1756-0500-5-596 (PMC3532230; doi:10.1186/1756-0500-5-596)
Supplement: Additional file 2 — User Guide. MetabR user guide. [file 1756-0500-5-596-S2.pdf]

MetabR User Guide

Data files

See Table 1 below for an example of a correct data file format, copied from our mouse data in Additional Files 5 and 6. One or two input data files may be used in each analysis, and may be TXT or CSV format. If data files contain commas (i.e. “2,3-diphosphoglycerate”) then TXT should be used, and if data files contain blank spaces (i.e. “Acetyl CoA”) then CSV should be used. If two files are analyzed together, they must have identical information in the first 3 columns, and all columns other than those containing response variables (i.e. metabolites) should have identical headings in both files. Columns with too few non-missing values will be skipped in the analysis. For a column not to be skipped, it must have at least two different treatment groups containing at least 3 non-missing metabolite values.

Column 1 must contain the heading "ID" and a unique identification in each row.

Column 2 must contain the heading "Subject" and contain names of the experimental units in the rows. A subject can be observed multiple times (i.e., on multiple days), in which case the same subject name would be entered in multiple rows. It is convenient to include the treatment group to which each subject belongs as part of the subject names so that it is easy to see in the heat map whether the subjects cluster within their treatment groups.

Column 3 must contain the heading "Group" and contain the names of the treatment groups to which each subject belongs. Treatment group names should be character strings, such as “Group1”, “Group2”, etc., not numeric values.

Any confounding variables begin in column 4 and use as many columns as needed. It is recommended to use non-numeric values (i.e. “Day1”, “Day2”, “Day3”) for factor variables containing classes or groups. Variables such as internal standard, run day, and tissue mass are placed here. As mentioned in paragraph 1, these column headings in two data files analyzed together must be identical; the data under the headings can be different, however, for instance if a different internal standard was measured in positive and negative mode.

Columns following the confounding variables contain measured metabolite values. These can be different between two data files analyzed together.

Missing values should be entered as “NA”.

Table 1. a and b. Shown here are excerpts of the two data files used in our mouse experiment (Additional Files 5 and 6). The column headings are identical up until the first metabolite column. The data in the first 3 columns are identical, while the “IS” column data are different because a different internal standard was used in positive and negative ion mode. “ID” contains a unique value for each row. “Subject” contains the names of the experimental units—mice, humans, tissue culture dishes, etc.—and Subject entries can be repeated. “Group” contains the treatment groups. ID and Subject may contain numerical or character strings, but Group must contain character strings. “Strain” here contains the two inbred strains of mice. “Day” contains the run days on which each sample was analyzed. “Quantity” contains the tissue mass analyzed. “IS” contains the internal standard measurements.

Random-effect variables like Strain and Day should have character string data, while fixed-effect variables like Quantity and IS can have character string or numeric data. Two Bisphenol A measurements were missing and so were coded as "NA". Additional metabolites would be put into columns continuing to the right.

a)

ID	Subject	Group	Strain	Day	Quantity	IS	Thymine	Urea
BPA50_C_mouse1_Day1	BPA50_mouse1	BPA50	C	Day1	6.95	4859047	5516	90999
BPA500_C_mouse2_Day1	BPA500_mouse2	BPA500	C	Day1	7.15	5238356	5185	69542
BPA5000_C_mouse3_Day1	BPA5000_mouse3	BPA5000	C	Day1	7.35	5257423	1957	130845
Control_C_mouse4_Day1	Control_mouse4	Control	C	Day1	12.85	5079308	8044	238918
BPA50_C_mouse5_Day1	BPA50_mouse5	BPA50	C	Day1	6.3	5239109	6392	107507
BPA500_C_mouse6_Day1	BPA500_mouse6	BPA500	C	Day1	14.65	5012908	5265	231653

b)

ID	Subject	Group	Strain	Day	Quantity	IS	Bisphenol A	Glucose-6-phosphate
BPA50_C_mouse1_Day1	BPA50_mouse1	BPA50	C	Day1	6.95	928146	1625	1446940
BPA500_C_mouse2_Day1	BPA500_mouse2	BPA500	C	Day1	7.15	878970	1318	1527127
BPA5000_C_mouse3_Day1	BPA5000_mouse3	BPA5000	C	Day1	7.35	1049784	NA	1424536
Control_C_mouse4_Day1	Control_mouse4	Control	C	Day1	12.85	856558	NA	1446332
BPA50_C_mouse5_Day1	BPA50_mouse5	BPA50	C	Day1	6.3	819029	1077	1467167
BPA500_C_mouse6_Day1	BPA500_mouse6	BPA500	C	Day1	14.65	807161	462	1512474

Running MetabR

Required

Install R version 2.15 by following the instructions at http://cran.r-project.org/.
Obtain the MetabR program file from http://metabr.r-forge.r-project.org/, and save it to your computer with the filename extension ".R".
Run R.
Under File, select "Change dir…" and select the folder containing the input data.
Under File, select "Source R code…" and select the MetabR program file.

When a window titled "CRAN mirror" opens, select the location closest to you. When a window titled "Repositories" opens, make sure that "BioC software" and anything already highlighted are selected.

Several required packages are automatically installed. Occasionally (particularly the first time running MetabR) an error occurs during this step. If this occurs, re-opening MetabR once or twice corrects the problem.

A window titled “MetabR—Data input” opens, shown in Figure 1 below. Select whether TXT or CSV files are used. Click “Data 1” to find and read in one data file. “Data 2” can be used to read in a second data file. Click “Clear Data 2” if you have selected a second data file but decide you want to only analyze one data file instead (such as after you have run MetabR). In “Column of first data measurement” enter the column number in which the first measured metabolite is found (“8” for the example data in Table 1). Select “OK”.

A second window titled “MetabR” appears, shown in Figure 2 below. The “Required” Tab must be used in every analysis. The other tabs control cosmetic aspects of plotting and significance thresholds for the Pathway Projector file and will use default settings unless changed. Under “Fixed-effect variables” and “Random-effect variables” in the Required tab, select the variables you want to define as fixed- and random-effect in the normalization model. Under “Output” you may tag output file names with a unique name. Under “Criterion”, select “P-val”, “Q-val”, “Fold-change”, “P-val+Fold-change”, or “Q-val+Fold-change” as the criteria for screening for significant metabolites. Under “Mean plot choice”, select whether p-values or q-values will be used as the criterion for generating significance letters to indicate which treatment groups differ significantly. Under “Log base” either accept a log base 2 data transformation, or change it; enter “1” for no transformation, or “exp(1)” or 2.718 for natural logarithm. Under “Fold-change cutoff”, accept the default threshold of mean fold-change > 1.5, or change it. Under “P or Q cutoff”, accept the default threshold of p-value or q-value < 0.05, or change it. Metabolites with any two treatment groups having a more extreme ANOVA Tukey HSD p-value or q-value and/or mean fold-change, depending on Criterion selection, are printed in the R console and plotted in mean plots.

Once the Required tab is set up correctly, select “Run”. The program may appear not to be running, but it runs as long as no error message is returned in the R console (warnings are normal), taking anywhere from 10 seconds (for our small example data files) to nearly 10 minutes for large proteomic data files with thousands of response variables and many treatment groups. The analysis is complete once several lists of metabolites are printed in the R console.

Running MetabR again

To run MetabR again, exit out of the “MetabR” window back to the “MetabR—Data input” window, select data files again, and proceed as before. If only 1 dataset will be used, use the “Data 1” button to select it, and hit “Clear Data 2”.

Optional

The tabs other than “Required” allow flexibility in plotting details. Experienced R users who desire a full explanation of the optional parameters can view them at the R help pages for functions shown in Table 2.

Others may refer to table 3 for information pertaining to all plots.

Pathway Projector

Pathway Projector is available at http://www.g-language.org/PathwayProjector/. In our experience, Internet Explorer does not work well with Pathway Projector, but Mozilla Firefox does. Select “Pathway Projector” from the top menu, and then select “Tools”. The “Browse” button allows you to select a file to upload from your computer desktop browser. In order to map metabolites, the official KEGG IDs or KEGG-recognized metabolite names must be used. These can be obtained by searching for the metabolite name at http://www.genome.jp/dbget-bin/www_bfind?compound. Serine, for example, would be replaced by “C00065”. You may either replace all metabolite names with these names in the

input data file(s), or replace the names in column 1 of the csv output file containing “colors” in the file name. Column 2 of this file contains coded values for the dot colors. Column 3 contains the size of the dots. Column 4 contains the p-values or q-values, and column 5 contains the font size of the p-value or q-value labels. These parameters can be controlled in the Pathway Projector tab in the MetabR menu. Upload this file, click “Generate Overlay Map”, and wait as Pathway Projector creates the map. Click on a dot to see information about the metabolite. If there is an error resulting from one of the rows of the file that is uploaded, the metabolite may not be mapped.

Output files

1) A CSV spreadsheet containing the normalized data.
2) A CSV spreadsheet containing the normalized data with technical replicate measurements averaged (this data matrix is used for statistical analysis).
3) A PDF file containing a plot of the model residuals for each metabolite vs. each metabolite’s overall mean signal. This plot is for all metabolites combined, sufficient for a rapid overall assessment of unequal variance. This plot also can be used to assess how well the log transformation (base 2 was chosen for this analysis) controlled the typical relationship of increasing variance with increasing mean.
4) A second PDF file containing the same residual plot, only labeled with metabolite names. This is redundant; however, we find that the plotting window must be extremely wide (and thus difficult to view) to fit many metabolite names without them overlapping, and users may find both useful).
5) A pdf file containing mean plots for all significant metabolites based on the user’s choice of significance threshold. Treatment group means (based on log transformed data, if selected) and 95% confidence interval bars are plotted. Mean fold-changes between all pairs of treatment groups are shown. Lastly, pairs of treatment groups with statistically significant Tukey HSD p-values or q-values (user-selected threshold) are distinguished by labeling the treatment groups with different letters of the alphabet. Fold-changes between group means (not log transformed) are displayed below the letters. Fold-changes in the first row correspond to comparisons with the group in the first column (Control), fold-changes in the second row correspond to comparisons with the group in the second column (Fast), and so on.
6) A CSV spreadsheet containing Tukey HSD p-values for all treatment group comparisons for every metabolite.
7) A CSV spreadsheet containing q-values for all treatment group comparisons for every metabolite.

8) A CSV spreadsheet containing mean fold-changes between all pairs of treatment groups for every metabolite.
9) A PDF file containing plots of all confounding variables vs. all metabolite measurements, pre- and post-normalization.
10) A PDF file containing a heat map and dendrogram of the normalized data generated using R function "heatmap.2" from package "gplots". A heat map is useful for visualizing overall differences in metabolic patterns, and the dendrogram drawn on the experiment samples gives visual evidence of whether or not the experimental conditions significantly influenced metabolic patterns. Each metabolite plotted is mean-centered, helping to call attention to metabolites differing in abundance between treatment groups. Missing values are indicated in white. If too many missing values are present, the heat map function would normally return an error. MetabR first tries to draw the heat map with missing values included. If an error would have been returned, the heat map is drawn with zeros substituted in place of missing values.
11) A CSV spreadsheet that can be directly uploaded to Pathway Projector to map metabolomic changes to metabolic pathways. The resulting pathway map contains dots of three shades of red and three shades of blue. The darkest colors indicate that the mean fold-change for a particular metabolite was greater than the "Threshold 1" value set in the Pathway Projector tab in the MetabR menu. The colors of medium and highest brightness indicate that thresholds 2 and 3 were passed, respectively. The user selects which 2 treatment groups to compare in the Pathway Projector tab; the fold-change here is calculated as Treatment/Control.

Table 2. Help page information for experienced R users who want a full explanation of how to control the parameters from the optional tabs. All parameters are arguments used with the functions and their associated packages shown in this table.

R function ("package")	Command in R Console
par ("graphics")	help(par)
plotmeans ("gplots")	help(plotmeans)
levene.test ("lawstat")	help(levene.test)
heatmap.2 ("gplots")	help(heatmap.2)

Table 3. Specific instructions for how to adjust plotting parameters in the optional tabs, such as font size, plotting window size, and labeling orientation.

What to change	Parameter to use	Instructions
Axis label orientation	las	Enter 0, 1,2, or 3, for parallel to axis, horizontal, perpendicular to axis, or vertical.
Axis annotation font size	cex.axis	Enter positive values 0 through 1 or

		greater than 1 to decrease or increase font size, respectively.
Axis label font size	cex.lab	Enter positive values 0 through 1 or greater than 1 to decrease or increase font size, respectively.
Main title font size	cex.main	Enter positive values 0 through 1 or greater than 1 to decrease or increase font size, respectively.
Mean plot data labels font size	cex(text)	Increase cex(text) to increase font size of significance letters, fold-change labels, and group mean labels
Height of plotting window	height	Default is 7. Increase height to prevent horizontal text labels in rows from overlapping.
Width of plotting window	width	Default is 7. Increase width to prevent vertical text labels in columns from overlapping.
Margins outside of plot	mar	Order is c(bottom, left, top, right). Increase margins to fit long text labels.
Heat map margins	margins	Order is c(vertical, horizontal). Increase vertical margin to fit long metabolite names oriented vertically, and increase horizontal margin to fit long subject labels oriented horizontally.

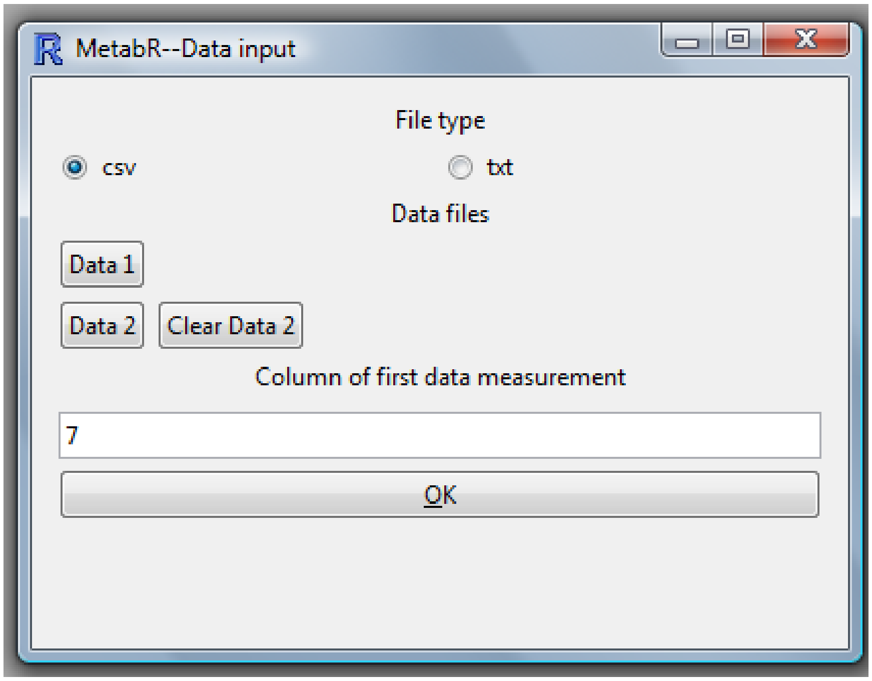

Figure 1. MetabR data input window.

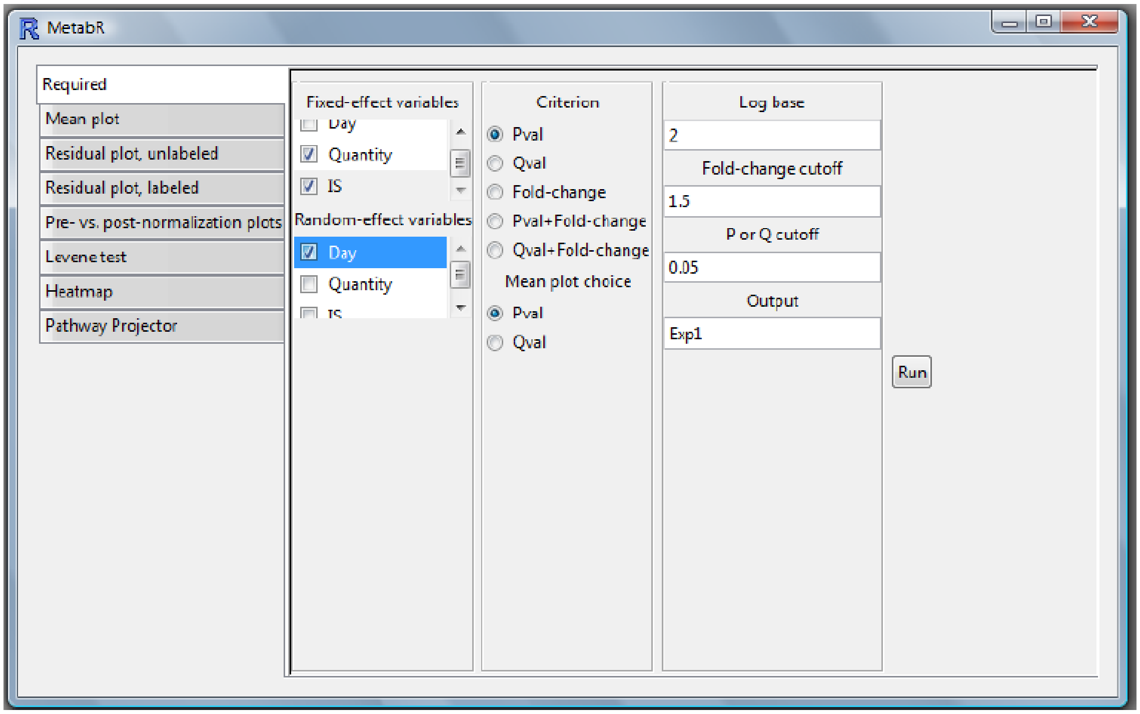

Figure 2. Main MetabR window.
